# Impact of resuscitation fluids in the pediatric emergency department on length of stay, electrolyte balance, and kidney function

**DOI:** 10.1007/s00431-025-06665-w

**Published:** 2025-12-01

**Authors:** Giora Weiser, Yael Siedner-Weintraub

**Affiliations:** 1 Pediatric Emergency Department, Shaare Zedek Medical Center, Jerusalem, Israel; 2 Pediatric Intensive Care Unit, Shaare Zedek Medical Center, Jerusalem, Israel; 3 Faculty of Medicine, Hebrew University in Jerusalem, Jerusalem, Israel

**Keywords:** Balanced fluids, Saline, Pediatric emergency, Sepsis, Fluid loss

## Abstract

The optimal resuscitation fluid in pediatric emergency departments remains unresolved. Balanced solutions, such as Ringer’s lactate, have emerged as alternatives to the long-standing use of normal saline; however, existing studies have not demonstrated significant differences in clinical outcomes. This study aimed to assess the clinical impact of transitioning from normal saline to Ringer’s lactate solution in the pediatric emergency department, primarily in terms of length of hospital stay, alongside kidney functions and biochemical measures. We conducted a prospective study comparing clinical outcomes of a transition from normal saline to Ringer’s lactate for resuscitation boluses. Each of the two study groups included 107 pediatric patients. A mean of 42 mL/kg was administered as a fluid bolus in the normal saline and Ringer’s lactate groups (42.04 vs 42.1 mL/kg respectively). The most common diagnosis was fluid losses, followed by sepsis (81% vs 83% and 13% vs 11% respectively). Notably, the length of stay was significantly shorter in the Ringer’s lactate group (2.6 days) compared to the normal saline group (3.4 days). No significant differences were observed in acid–base balance, electrolyte levels, or kidney function indicators. These findings were consistent among a subset of patients with acute kidney injury at admission.

* Conclusions*: The use of Ringer’s lactate resulted in a shorter length of hospital stay, with implications for both medical care and healthcare costs. No other significant differences were identified between the two fluid solutions.

**Table:** 

**What is Known:**
• *Fluid boluses are the mainstay of treating children with signs of shock. The use of balanced fluids has not shown an advantage relating to morbidity or mortality. Most pediatric emergency departments use normal saline as a standard, while intensive care units shift to balanced solutions*.
**What is New:**
• *Using a balanced solution as the fluid of choice for boluses in a pediatric emergency department was feasible and showed a streamlined fluid treatment. The use of a balanced solution from emergency department presentation was associated with a shorter length of stay*.

## Introduction

Fluid resuscitation in the pediatric population primarily relies on isotonic fluids [1]. Historically, 0.9% sodium chloride (normal saline, NS) has been the fluid of choice [2]. However, due to its super-physiologic chloride concentration, concern has risen regarding its potential to worsen acidosis and cause acute kidney injury (AKI). An alternative to NS, balanced crystalloids (BC), such as Ringer’s lactate (RL), solutions are isotonic and contain less chloride, making them appear more physiologic. Studies on adults comparing RL and NS have demonstrated improved outcomes with RL, including reduced mortality, lower incidence of AKI, and fewer electrolyte imbalances [3]. However, such findings remain inconclusive in pediatric populations, as studies are limited.

Several studies on pediatric patients have explored fluid resuscitation outcomes in contexts such as sepsis, diarrhea and dehydration, asthma, various surgical interventions, and general bolus use [4–10]. These studies, conducted in various settings (e.g., emergency departments (ED), intensive care units, and operating rooms), did not reveal significant differences in mortality, AKI, or blood electrolyte imbalance. Systematic reviews also failed to find conclusive differences [11]. A meta-analysis published in 2024 [12] reported that using BC fluids was associated with reduced mortality and AKI. However, this meta-analysis included only four randomized controlled trials out of eight reviewed, and only one of these trials had a large sample size population to demonstrate significant differences.

In our tertiary medical center, NS was the fluid of choice in the pediatric ED (PED) for fluid resuscitation until 2021, when, in an attempt to standardize fluid therapy for all patients, RL became the primary resuscitation fluid. This process was an institutional collaboration to standardize fluid resuscitation in pediatric patients. It followed a joint decision by the PED and pediatric intensive care (PICU) physicians. In this prospective study, we investigated clinical outcomes, primarily length of hospital stay (LOS), incidence of AKI, and biochemical measures such as acid–base balance and other electrolyte disturbances.

## Methods

### Study design

We conducted an interrupted time series study of all patients who received fluid resuscitation at our PED. Data was collected during two time periods: January 1, 2018, to December 31, 2019, when NS was the primary resuscitation fluid (NS cohort), and March 1, 2022, to February 28, 2024, when RL became the primary fluid (RL cohort). Data collection between 2020 and early 2021 was excluded due to the SARS-CoV-2 pandemic.

Data were collected retrospectively for the 2018–2019 cohort and prospectively for the 2022–2024 cohort. The study was approved by the hospital’s institutional review board (Helsinki SZMC-21–0198), which also waived the requirement for informed consent.

### Population

The cohorts included all patients aged 0–18 years who were admitted to the ED and resuscitated with 30 ml or more of fluids per kilogram. We excluded patients with diabetic ketoacidosis, traumatic brain injury, or hyponatremia, as treatment protocols for these conditions require the use of NS.

### Data collection

Data were extracted from electronic health records (EHR). The following information was collected:

- **Demographics**: Age, sex, and weight at admission.
- **Clinical Information**: Indication for fluid resuscitation (e.g., fluid losses from vomiting, diarrhea, poor intake), sepsis or septic shock, acute kidney injury (AKI) defined as creatinine levels above age-specific normal limits [13], vital signs, amount of fluids administered per kilogram, department of admission from PED, length of stay, and need for oxygen or respiratory support (e.g., high-flow nasal cannula or intubation).
- **Biochemical results**: (from the blood draw taken on arrival at the PED)
  o Values on admission: pH, pCO_2_ (mmHg), HCO_3_⁻ (mmol/L), sodium (mEq/L), potassium (mEq/L), chloride (mEq/L), creatinine (mg/dL), and blood urea nitrogen (BUN, mg/dL).
  o Minimal pH, pCO_2_, HCO_3_^−^, and BUN during admission.
  o Maximal sodium, potassium, chloride, creatinine, and BUN during admission.

### Outcomes

The primary outcome was the length of hospital stay, being the more significant outcome in the current literature. Secondary outcomes included differences in acidosis, renal functions (adjusted for age), and metabolic abnormalities. These outcomes were evaluated for the entire cohort and, in addition, for a subset of patients from both cohorts who had AKI upon admission to PED.

### Statistical analysis

#### Matching

We matched patients in the treatment groups on a one-to-one basis using k-nearest neighbors without replacement and a caliper of 0.15 on the propensity score. Exact matching on the department of admission was also applied to improve group balance. Variables used for matching included age, sex, weight, indication for fluid resuscitation, fluid dose, and biochemical results on admission to ED (BUN, sodium, and creatinine). Baseline covariate balance was assessed using standardized mean differences (SMD), with SMD > 0.25 indicating significant imbalance.

#### Statistical methods

Continuous descriptive data are presented as means ± standard deviation (SD) or medians (interquartile range), while categorical data are presented as frequencies and percentages. Groups were compared using Student’s *t*-test, Wilcoxon rank-sum test, *χ*^2^ test, or Fisher’s exact test, as appropriate. Adjusted means were calculated for each group to interpret main effects, and post hoc pairwise comparisons were conducted using Bonferroni correction to control for Type I error. A *p*-value < 0.05 was considered significant. All analyses were performed using RStudio version 4.3.2.

#### Ethics approval

This study was approved by the SZMC review board in accordance with the Declaration of Helsinki (SZMC-21–0198).

## Results

**Fig. 1:**
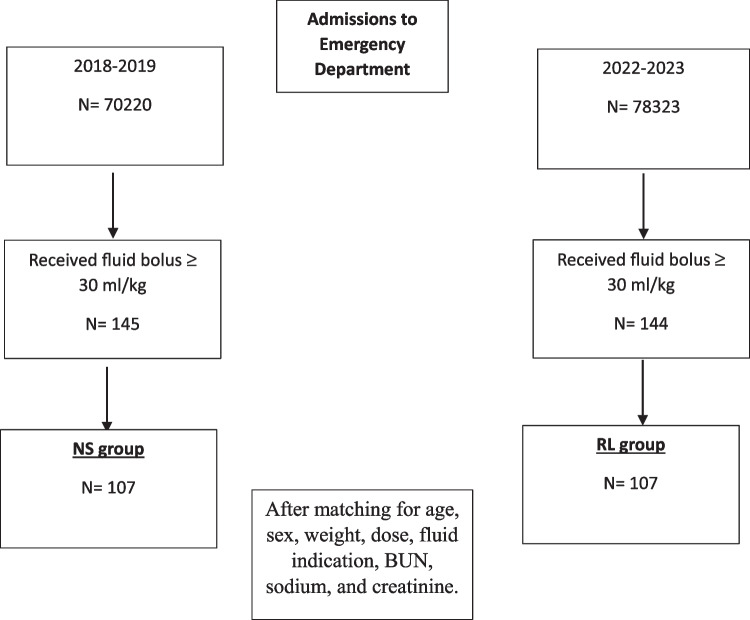
Study flow chart

A total of 289 patients received fluid resuscitation in the PED during the study periods: 145 patients in the 2018–2019 cohort (NS cohort) and 144 in the 2022–2023 cohort (RL cohort). After matching, 107 patients remained in each cohort (Fig. 1). Blood gas analysis was not performed on admission for 37 patients in the NS cohort and 36 in the RL cohort.

The mean amount of fluid administered for resuscitation was 42 mL/kg (SD 7.8) in the NS group and 42 mL/kg (SD 9.96) in the RL group (*p* = 0.965).

The most common diagnosis in both groups was fluid losses (i.e., dehydration) followed by sepsis and other causes.

Demographics and clinical baseline characteristics are summarized in Table 1. Patients in both groups had similar characteristics.

**Table 1:** Demographics and clinical characteristics on admission in matched cohorts

Characteristics	NS-2018–2019 (*N* = 107)	RL-2022–2024 (*N* = 107)	*p*	SMD
Age (mean (SD))	4.72 (5.55)	4.36 (5.08)	0.621	0.068
Male (%)	53 (49.5)	53 (49.5)	1	< 0.001
Weight (kg) (median [IQR])	10.30 [6.83, 22.75]	9.60 [7.16, 19.25]	0.806	0.037
Admitting department (%)			1	< 0.001
General pediatrics	83 (77.6)	83 (77.6)		
Surgical	13 (12.1)	13 (12.1)		
Critical care	11 (10.3)	11 (10.3)		
Fluid bolus volume (mL/kg) (mean (SD))	42.04 (7.83)	42.10 (9.96)	0.965	0.006
Indication for fluid bolus (%)			0.979	0.06
Fluid loss	87 (81.3)	89 (83.2)		
Sepsis and septic shock	13 (12.1)	11 (10.3)		
Acute kidney injury	1 (0.9)	1 (0.9)		
Other	6 (5.6)	6 (5.6)		

*SD* standard deviation, *IQR* interquartile range, *SMD* standardized mean differences

Clinical and biochemical findings on admission are compared in Table 2. None of the findings had a significance or clinical impact.

**Table 2:** Biochemical results on admission in matched cohorts

Characteristics	Normal saline 2018–2019 (*N* = 107)	Ringer’s lactate 2022–2024 (*N* = 107)	*p*	SMD
pH (mean (SD))	7.32 (0.11)	7.31 (0.12)	0.562	0.098
pCO_2_ (mmHg) (mean (SD))	32.57 (10.18)	32.98 (7.51)	0.786	0.046
HCO_3_^−^ (mmol/L) (mean (SD))	18.37 (6.14)	17.55 (6.73)	0.455	0.126
Potassium (mEq/L) (mean (SD))	4.27 (1.12)	4.09 (0.70)	0.149	0.199
Sodium (mEq/L) (mean (SD))	138.05 (8.95)	137.80 (5.97)	0.816	0.032
Chloride (mEq/L) (mean (SD))	109.75 (12.48)	107.89 (10.34)	0.333	0.163
Creatinine (mg/dL) (mean (SD))	0.51 (0.37)	0.47 (0.45)	0.439	0.106
BUN (mg/dL) (mean (SD))	17.71 (13.44)	17.07 (12.51)	0.721	0.049

^*^BUN blood urea nitrogen, *SD* standard deviation, *SMD* standardized mean differences

Patients in the RL cohort had a significantly shorter length of stay (2.6 days vs. 3.4 days, *p* = 0.01).

Biochemically, the RL cohort showed a significantly lower maximal potassium level (4.3 mEq/L vs. 4.6 mEq/L, *p* = 0.007). Changes in pH and bicarbonate (HCO_3_⁻) were greater in the NS cohort than in the RL cohort (pH—median interquartile range [IQR]: 0.00 [0.00, 0.04] vs. 0.00 [0.00, 0.00], *p* = 0.018; HCO_3_⁻—median [IQR]: 0.00 [0.00, 2.4] vs. 0.00 [0.00, 0.00], *p* = 0.003). However, the magnitude of these differences was minimal. No significant differences were observed in pCO_2_, electrolytes, or kidney function measures. These are summarized in Table 3.

**Table 3:** Clinical and biochemical outcomes

Outcomes	Normal saline 2018–2019 (*N* = 107)	Ringer’s lactate 2022–2024 (*N* = 107)	*p*
Length of stay (days) (median [IQR])	3.40 [2.08, 5.72]	2.61 [1.67, 4.32]	0.01
Minimal pH (mean (SD))	7.29 (0.13)	7.29 (0.13)	0.979
Minimal HCO_3_^−^ (mmol/L) (mean (SD))	17.09 (6.07)	17.00 (5.82)	0.93
Maximal potassium (mmol/L) (mean (SD))	4.59 (1.10)	4.26 (0.61)	0.007
Maximal sodium (mmol/L) (mean (SD))	141.96 (8.39)	140.83 (5.79)	0.253
Maximal chloride (mmol/L) (mean (SD))	113.30 (11.43)	111.67 (9.08)	0.346
Maximal creatinine (mg/dL) (median [IQR])	0.40 [0.29, 0.63]	0.38 [0.27, 0.50]	0.143
Maximal BUN (mg/dL) (median [IQR])	13.00 [9.00, 23.00]	13.00 [8.50, 20.00]	0.761
Change in pH (median [IQR])	0.00 [0.00, 0.04]	0.00 [0.00, 0.00]	0.018
Change in HCO_3_^−^ (median [IQR])	0.00 [0.00, 2.40]	0.00 [0.00, 0.00]	0.003
Change in potassium (median [IQR])	0.00 [− 0.40, 0.00]	0.00 [− 0.30, 0.00]	0.362
Change in sodium (median [IQR])	− 1.00 [− 7.00, 0.00]	0.00 [− 4.00, 0.00]	0.299
Change in chloride (median [IQR])	0.00 [− 6.00, 0.00]	0.00 [− 5.00, 0.00]	0.885
Change in creatinine (median [IQR])	0.00 [0.00, 0.00]	0.00 [0.00, 0.00]	0.345
Change in BUN (median [IQR])	0.00 [0.00, 0.00]	0.00 [0.00, 0.00]	0.448

In a subset analysis of patients with AKI, those in the RL cohort had an apparent shorter length of stay than the NS cohort (2.9 days vs. 4 days), although this did not reach statistical significance. Biochemically, the RL cohort had a significantly lower maximal potassium level than the NS cohort (4.3 mEq/L vs. 4.7 mEq/L, *p* = 0.042). Changes in bicarbonate were higher in the NS cohort than in the RL cohort (HCO_3_⁻—median [IQR]: 0.00 [0.00, 3.2] vs. 0.00 [0.00, 0.00], *p* = 0.029). These findings are presented in Table 4.

**Table 4:** Clinical and biochemical outcomes for patients with abnormal baseline kidney function

Outcomes	NS- 2018–2019 (*N* = 32)	RL-2022–2024 (*N* = 35)	*p*
length of stay (days) (median [IQR])	4.02 [2.05, 7.23]	2.89 [2.00, 4.37]	0.132
alive = 1 (%)	1 (3.1)	0 (0.0)	0.964
pH min (mean (SD))	7.26 (0.17)	7.29 (0.13)	0.490
pCO_2_ max (mean (SD))	44.43 (21.38)	39.82 (8.05)	0.283
HCO_3_^−^ min (mean (SD))	16.54 (5.14)	17.47 (5.58)	0.518
Potassium max (mean (SD))	4.73 (1.01)	4.32 (0.60)	0.042
Sodium max (mean (SD))	146.25 (12.71)	143.57 (7.10)	0.285
Chloride max (mean (SD))	116.75 (13.77)	113.55 (8.40)	0.280
Creatinine max (median [IQR])	1.03 [0.64, 1.43]	0.77 [0.62, 1.33]	0.280
BUN max (median [IQR])	34.00 [23.50, 42.25]	33.00 [23.00, 44.50]	0.950
pH diff (median [IQR])	0.00 [0.00, 0.07]	0.00 [0.00, 0.00]	0.068
pCO_2_ diff (median [IQR])	− 5.85 [− 11.10, 0.00]	− 6.20 [− 9.60, 0.00]	0.550
HCO_3_^−^_diff (median [IQR])	0.00 [0.00, 3.17]	0.00 [0.00, 0.00]	0.029
Potassium_diff (median [IQR])	− 0.20 [− 0.70, 0.00]	0.00 [− 0.45, 0.00]	0.157
Sodium_diff (median [IQR])	− 5.00 [− 9.00, − 1.00]	− 3.00 [− 7.50, − 1.00]	0.337
Chloride_diff (median [IQR])	− 5.50 [− 10.25, 0.00]	− 2.00 [− 10.50, 0.00]	0.663
Creatinine_diff (median [IQR])	0.00 [0.00, 0.00]	0.00 [0.00, 0.00]	0.271
BUN_diff (median [IQR])	0.00 [0.00, 0.00]	0.00 [0.00, 0.00]	0.811

There were no cases of mortality in the study groups.

## Discussion

The debate surrounding the choice of resuscitation fluid in children has been extensively studied. Recent reviews show no difference in mortality or other major outcomes [4, 12]. Typically, a shorter LOS is observed in patients treated with RL or other balanced solutions. This study suggests that both NS and RL are safe and effective for pediatric fluid resuscitation.

Our study contributes to the existing literature by prospectively examining fluid resuscitation across various causes. We did not find significant differences in mortality, AKI, or biochemical outcomes, consistent with previous studies.

Gastroenteritis and dehydration were the most common indications for fluid resuscitation, with sepsis accounting for approximately 11–13% of patients in both study groups. While RL is often associated with better outcomes in cases of sepsis, the relatively low incidence of sepsis in our study limits the ability to draw definitive conclusions. The lower severity of illness, as indicated by a small proportion of patients requiring admission to the PICU, again limits the ability to separate the outcomes.

This study provides additional evidence that using RL as the choice resuscitation fluid is associated with a shorter LOS. Although an explanation for this remains unclear, it is plausible that a more balanced solution may offer a more efficient recovery by a yet unresolved pathway. This has been previously shown in a large propensity matched database in pediatric sepsis [14]. Further investigation in larger multi-center studies is warranted. Results from an ongoing multicenter study may provide a clearer picture (PROmPT bolus study) [15].

Notably, a shorter LOS is beneficial not only for patient outcomes but also for the financial aspects of care. This has important implications for institutional decisions regarding the choice of resuscitation fluid in the pediatric emergency department. Regardless, a shorter LOS is undoubtedly in the best interest of the medical outcome of patients.

Additionally, no significant differences were found in kidney function outcomes, further suggesting that the choice of fluid does not impact AKI recovery.

Historically, NS has been the standard resuscitation fluid in pediatric emergency care, despite other departments and intensive care units favoring balanced solutions like RL. Our study demonstrates that transitioning to RL as the primary resuscitation fluid was successful, creating consistency in fluid management protocols across the hospital. While there was no clear clinical advantage for RL over NS, the switch to use RL facilitated streamlined patient care across departments. This was important in creating clear institutional guidelines. The financial implications of this change are significant and should not be overlooked.

This study is limited by its relatively small cohort size and insufficient power to draw clear conclusions about the biochemical differences which were noted but non-significant. The lack of data on illness severity hampers the ability to clarify the effect of fluid resuscitation. This was based mostly on the need for PICU admission. There was missing information as to fluid volumes received prior to PED admission. In addition, differences in LOS may be affected not only by fluid type but also by hospital practices in the different study periods.

## Conclusion

The use of RL in the PED is feasible and is associated with a shorter LOS, which may have important medical and financial implications. No other significant differences in patient outcomes between the two treatment groups were observed. This study contributes to the growing body of literature focused on identifying the best resuscitation fluid for pediatric patients.

## Data Availability

The data from this study is available upon reasonable request.

## Abbreviations

AKI: Acute kidney injury
NS: Normal saline
RL: Ringer’s lactate
BC: Balanced crystalloids
PED: Pediatric Emergency Department
LOS: Length of stay
EHR: Electronic health record
PICU: Pediatric intensive care unit
